# Evaluation of osseointegration of plasma treated polyaryletherketone maxillofacial implants

**DOI:** 10.1038/s41598-024-80335-z

**Published:** 2025-01-13

**Authors:** D S Abdullah Al Maruf, Jiongyu Ren, Kai Cheng, Hai Xin, Will Lewin, Edmund Pickering, Hedi Verena Kruse, David Leinkram, Krishnan Parthasarathi, Innes Wise, Benjamin Filippi, Stephen Beirne, Cate Froggatt, James Wykes, Dale Howes, Natalka Suchowerska, Maria A. Woodruff, Jeremy M. Crook, David R. McKenzie, Jonathan R. Clark

**Affiliations:** 1 Central Clinical School, Faculty of Medicine and Health, The University of Sydney, Camperdown, Australia; 2 Integrated Prosthetics and Reconstruction, Department of Head and Neck Surgery, Chris O’Brien Lifehouse, Camperdown, Australia; 3 School of Mechanical, Medical and Process Engineering, Queensland University of Technology, Brisbane, Australia; 4 Central Analytical Research Facility, Research Infrastructure, Queensland University of Technology, Brisbane, Australia; 5 ARC Training Centre for Cells and Tissue Engineering Technologies, Queensland University of Technology, Brisbane, Australia; 6 Royal Prince Alfred Institute of Academic Surgery, Sydney Local Health District, Camperdown, Australia; 7 Arto Hardy Family Biomedical Innovation Hub, Chris O`Brien Lifehouse, Camperdown, Australia; 8 Laboratory Animal Services, The University of Sydney, Camperdown, Australia; 9 AIIM Facility, Intelligent Polymer Research Institute, The University of Wollongong, Wollongong, Australia; 10 School of Physics, Faculty of Science, The University of Sydney, Camperdown, Australia; 11 School of Dentistry, Faculty of Medicine, University of Sydney, Camperdown, Australia; 12 School of Medical Sciences, Faculty of Medicine and Health, The University of Sydney, Camperdown, Australia; 13 Sarcoma and Surgical Research Centre, Chris O’Brien Lifehouse, Camperdown, Australia

**Keywords:** Plasma ion immersion implantation, Polyether ether ketone, PEEK, Polyether ketone, PEK, Mandible, Maxilla, Bone implant, Osseointegration, Biotechnology, Medical research

## Abstract

Osseointegration is a crucial property of biomaterials used for bone defect repair. While titanium is the gold standard in craniofacial surgeries, various polymeric biomaterials are being explored as alternatives. However, polymeric materials can be bioinert, hindering integration with surrounding tissues. In this investigation, plasma ion immersion implantation (PIII)-treated polyether ether ketone (PEEK) and polyether ketone (PEK) implants were assessed in a sheep maxilla and mandible model. Defects were filled with PIII-treated PEEK and PEK implants, produced through fused filament fabrication (FFF) and selective laser sintering (SLS), respectively. Positive controls were grade 23 titanium implants via selective laser melting, while untreated PEEK implants served as negative controls. Surface analyses using scanning electron microscopy and atomic force microscopy revealed favorable properties. Osseointegration was qualitatively and quantitatively assessed at 8-, 10-, and 12-weeks post-implantation, showing significantly improved outcomes for both PIII-treated PEEK and PEK implants compared to untreated controls. The study suggests PIII treatment enhances FFF-printed PEEK’s osseointegration, and PIII-treated SLS-printed PEK achieves comparable osseointegration to 3D printed titanium. These findings underscore surface modification strategies’ potential for polymeric biomaterials, offering insights into developing alternative implant materials for craniofacial surgeries, with enhanced biocompatibility and osseointegration capabilities for improved clinical outcomes.

## Introduction

Segmental mandibular defects are a common problem faced in the treatment of benign and malignant tumours of the jaw and oral cavity^1^. Currently, vascularized bone flaps are routinely used to reconstruct these defects; however, these are long and complex operations that are associated with substantial donor site morbidity^2^. The complex geometry of the maxilla and mandible make these prime candidates for bone reconstruction using additive manufacturing with an appropriate bone-substitute biomaterial. Titanium has been widely applied in maxillofacial reconstruction, including the fixation of autologous bone, osseointegrated dental implants, and the replacement of selected bone defects^3–6^. It has the advantages of being strong, malleable, non-toxic, and it integrates with bone. Unfortunately, titanium also has several drawbacks in the setting of radical cancer treatment where radiotherapy dose intensification and poorly suited mechanical properties can lead to implant failure. Furthermore, X-ray dose shielding may increase the risk of tumour recurrence or delay its detection due to imaging artefacts.

Titanium has an elastic modulus (110 GPa)^7^ several times higher than that of cortical (18–20 GPa)^8–10^ or cancellous bone (10–14 GPa)^11^. This modulus mismatch is problematic for implants with high cantilever forces, such as those observed in the body of the mandible, where stress shielding and concentration may lead to bone resorption and gradual implant loosening^12^. This contrasts with other scenarios where axial loading of titanium implants is associated with favourable outcomes, such as dental implants^2^. Biomaterials used to bridge segmental defects repair should ideally possess some fundamental properties, including osteoinduction (a process that stimulates new bone formation), osteoconduction (formation of bone on the surface of a material), and osseointegration (a biological phenomenon resulting in the stable anchorage of an implant to the bone which can be quantified mechanically and/or by the degree of bone-implant contact)^2,13^. Osseointegration is determined by the surface properties of the implant, including hydrophilicity of the implant material and surface topology^14,15^.

Polyether ether ketone (PEEK) and polyether ketone (PEK) are members of the polyaryletherketone (PAEK) family that possess unique features such as radiolucency, non-reactivity, and temperature resistance^16^. The biomaterials of PAEK family have a relatively better elastic modulus (3.7–5.1 GPa)^2^ match to that of cortical bone when compared with titanium. The intrinsic radiolucency of PEEK and/or PEK confers a distinct advantage over titanium implants in craniomaxillofacial reconstruction by mitigating issues associated with x-ray scattering. This property enhances the clarity of radiographic evaluations, thereby improving the precision of clinical assessments^17^. PEEK has been used in the form of patient-specific custom implants for craniomaxillofacial reconstruction, but unlike titanium, it does not naturally osseointegrate or osteoinduce because it is hydrophobic and bioinert^18^. Various physical and chemical surface modification strategies have been developed to make PEEK more hydrophilic^19^. For example, plasma ion immersion implantation (PIII) treatment is a surface modification strategy that increases the hydrophilicity of PEEK^20^. We have shown that PIII-treatment of 3D-printed PEEK increases its hydrophilicity and subsequent bone mineralisation in vitro^21^ and the osseointegration of PIII-PEEK in vivo, using a flat torque implant mounted on the surface of the ovine scapula^22^. Notwithstanding our published work, whether or not PIII-treatment increases osseointegration when an implant is placed within living bone remains to be determined. In this pilot study, we implanted 3D-printed PEEK and PEK cylindrical structures treated with PIII into sheep mandibles and maxillae, comparing them to titanium implants as a positive control and untreated PEEK as a negative control. We hypothesize that the PIII surface modification will significantly improve bone-implant contact in the PEEK and PEK implants compared to untreated PEEK and titanium. To evaluate this, we aim to conduct histological analyses of the PIII-treated 3D-printed PEEK and PEK implants. In this pilot study, we implanted PIII-treated, 3D-printed cylindrical structures of PEEK and PEK into sheep mandibles and maxillae, using titanium implants as a positive control and untreated PEEK as a negative control. We hypothesized that PIII surface modification would significantly enhance bone-implant contact in the PEEK and PEK implants compared to both untreated PEEK and titanium. To assess this, we aimed to perform histological analyses on the PIII-treated 3D-printed PEEK and PEK implants.

## Results

A total of 56 cylinders were surgically implanted and 46 were available for analysis. The type, location, and duration of implantation are summarized in Supplementary Table 2.

### Scanning electron microscopy (SEM) and atomic force microscopy (AFM)

**Fig. 1:**
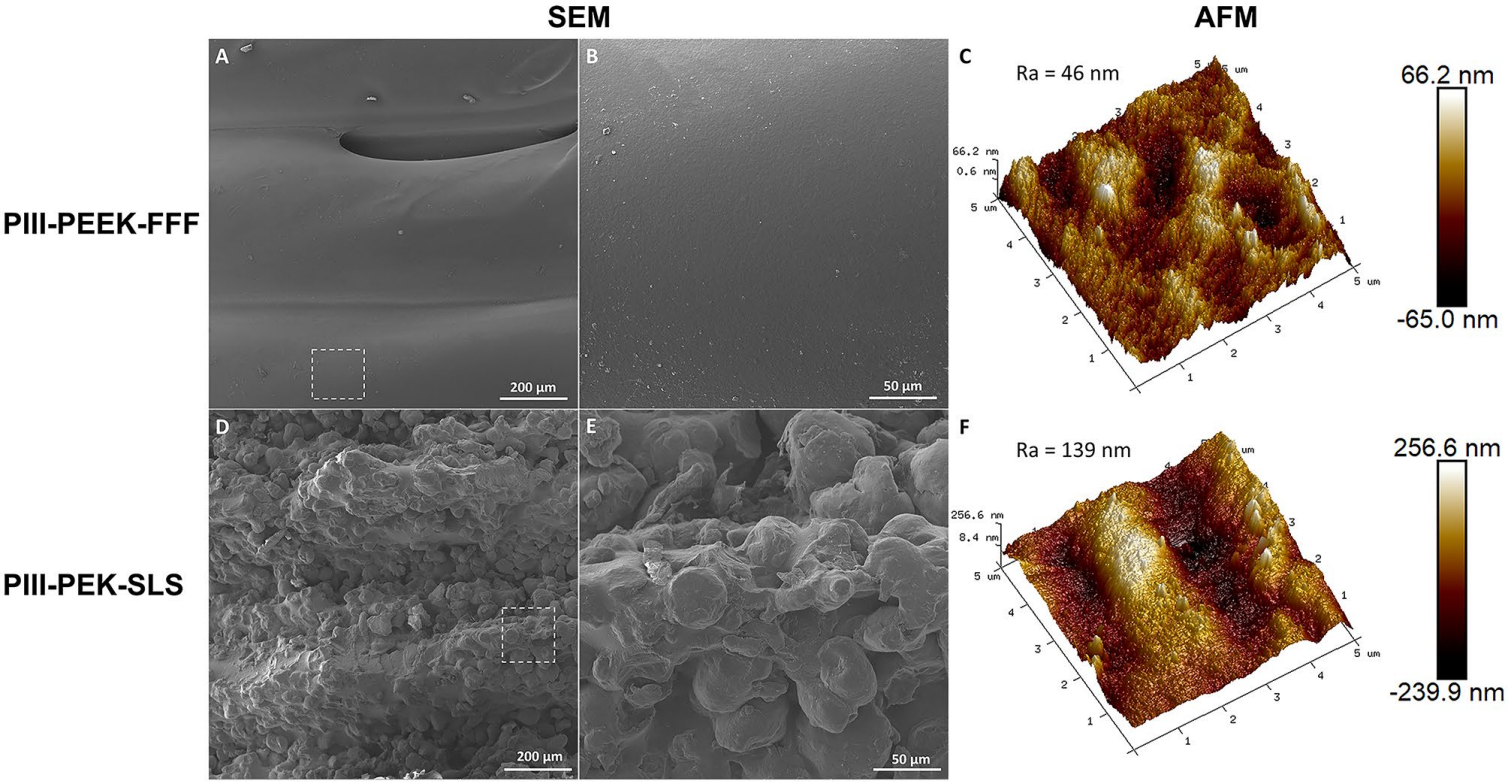
Representative SEM images of screw surfaces of PIII-PEEK-FDM (**A** and **B**) and PIII-PEK-SLS implants (**D** and **E**). Images (**B**) and (**E**) are high-magnification images of areas highlighted in the boxes in (**A**) band (**D**), respectively. The surface of PIII-PEK-SLS was visibly rougher than that of the PIII-PEEK-FDM. Image C and D are AFM images of PIII-PEEK-FDM and PIII-PEK-SLS respectively. Ra (the arithmetic average of the absolute values of the surface height deviations measured from the mean plane) was used to quantify the surface roughness and the Ra value was higher in the PIII-PEK-SLS surface compared to that of the PIII-PEEK-FDM surface.

Both PIII treated PEEK-FFF and PEK-SLS implants were imaged with SEM in the saw-tooth regions. As shown in Fig. 1A and B, the PIII-PEEK-FFF implant demonstrated a smooth surface topography when compared to the PIII-PEK-SLS implant as shown in Fig. 1D and E. In Fig. 1A, the FFF print layers are clearly visible. In contrast, the PIII-PEK-SLS implant had a more distinguishable saw-tooth structure (Fig. 1D and F). Consistent with the SEM images, the surface roughness of the PIII-PEK-SLS implants was higher (Ra = 139 nm) compared to the PIII-PEEK-FFF implant (Ra = 46 nm) (Fig. 1C and F).

### Histology stains and bone-implant contact (BIC) analysis

**Fig. 2:**
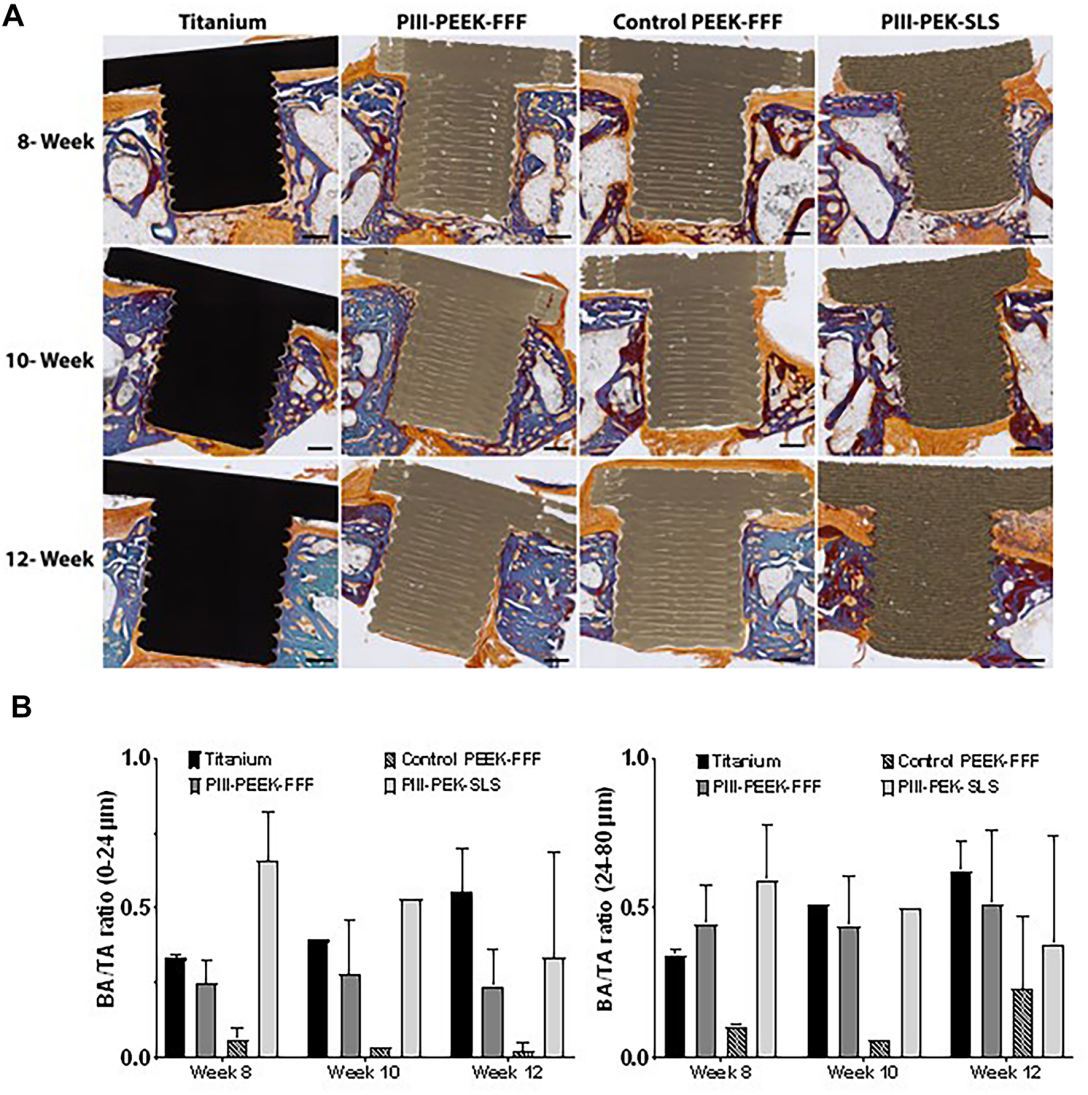
Histological analysis of bone-implant contact in mandible. (**A**) Representative images of the bone-implant interface in the mandible implant groups at 8-, 10- and 12-week time points. The scale bar = 1000 μm. One representative implant from each group and the surrounding tissues stained with Goldner’s trichrome are shown. The green/blue colour shows the mineralized bone, the red colour shows the mineralizing bone matrix, and the orange colour shows the collagen-rich connective tissues. The titanium implants and PEEK/PEK implants are black and brown, respectively. (**B**) Quantitative analysis of the BIC based on histology staining images in the mandible implant groups at 8-, 10- and 12-week time points. Results show mean ± standard error of the mean.

**Fig. 3:**
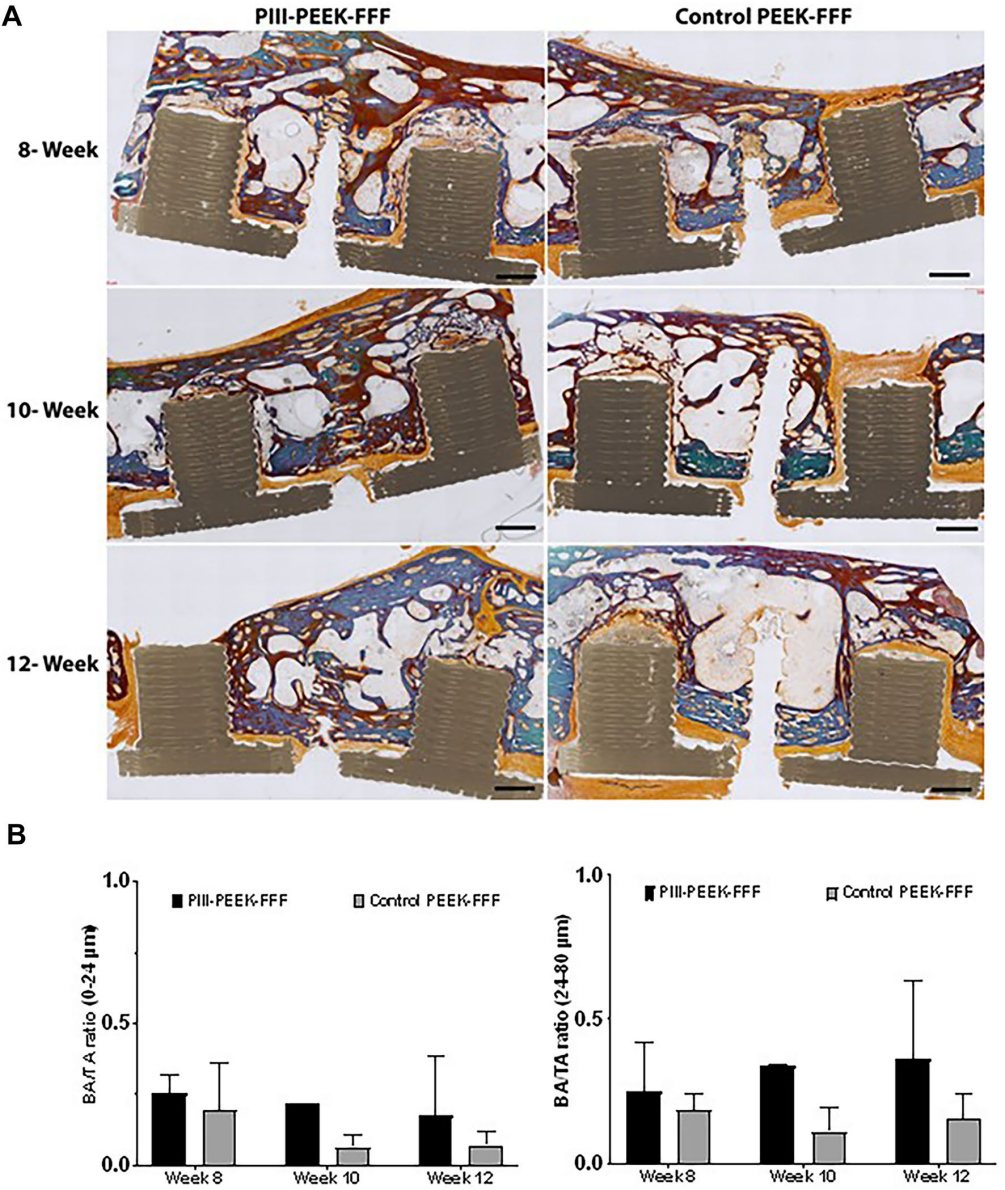
Histological analysis of bone-implant contact in maxilla. (**A**) Representative images of bone-implant interface in the maxilla implant groups at 8-, 10- and 12-week time points. The scale bar = 1000 μm. Two implants presented in the host bone and the surrounding tissues were stained with Goldner’s trichrome stain. (**B**) Quantitative analysis of the Bone implant Contact (BIC) based on histology staining images in the maxilla implant groups at 8-, 10- and 12-week time points. Results show mean ± standard error of the mean.

Figure 2A (mandible) and Fig. 3A (maxilla) show the histology of the implants and surrounding tissues. In Fig. 2A, both PIII-PEEK-FFF and PIII-PEK-SLS groups showed good bone-implant contact, similar to the titanium positive control. In contrast, the untreated control PEEK-FFF negative control had more connective tissue present on the implant surface. PIII-PEEK-FFF implants in the maxilla demonstrated similar results (Fig. 3A). The BA/TA results are shown in Figs. 2B and 3B for the mandible and maxilla implants, respectively. In the mandible, the plasma-treated PEEK-FFF and PEK-SLS implants demonstrated comparable BA/TA to that of the titanium implants at all time points in both 0–24 μm and 24–80 μm zones where the untreated control PEEK-FFF group showed lower BA/TA compared to all other implants in both zones (Fig. 2B). In the maxilla, the plasma-treated PEEK-FFF implants showed higher BA/TA compared to the untreated control PEEK-FFF implant control group at all time points in both 0–24 μm and 24–80 μm zones (Fig. 3B).

**Fig. 4:**
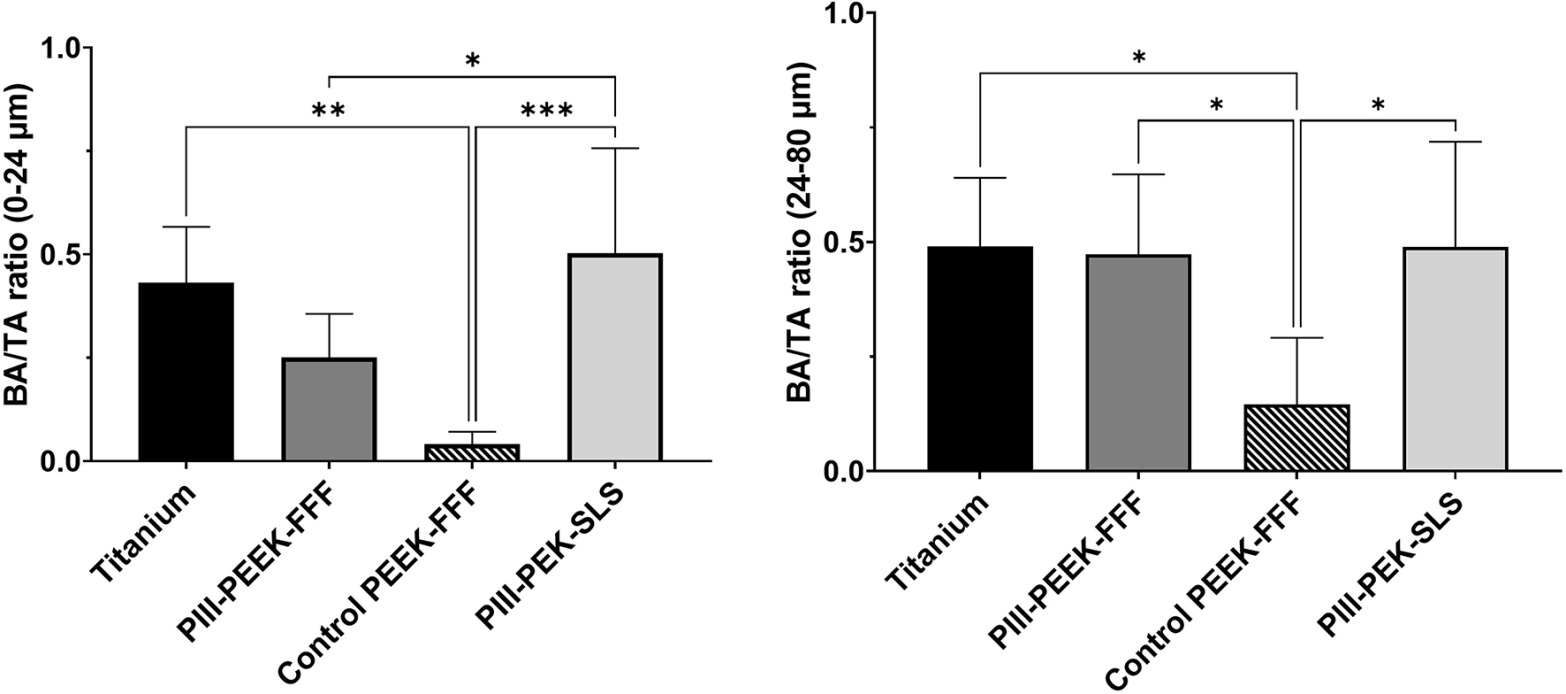
Analysis of the bone-implant contact (BIC) based on histology staining images in the four types of mandible implants at all time points combined (PIII-treated PEEK *n* = 10; PIII-treated PEK *n* = 5; Untreated PEEK *n* = 5; Titanium *n* = 5) Results show mean ± standard error of the mean. * indicates *p* < 0.05, ** indicates *p* < 0.01, and *** indicates *p* < 0.001.

**Fig. 5:**
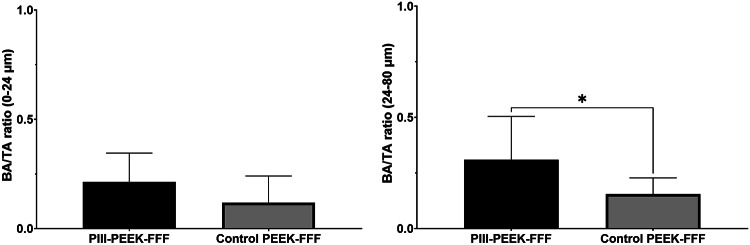
Analysis of the Bone implant Contact (BIC) based on histology staining images in the two types of maxilla implants at all time points combined (PIII-treated PEEK *n* = 10; Untreated PEEK *n* = 10). Results show mean ± standard error of the mean. * Indicates a *p* < 0.05.

Combining all time points there was no significant difference in BA/TA between the mandibular PIII-PEEK-FFF and titanium implants; in contrast, the untreated control PEEK-FFF implants showed significantly lower BA/TA compared to the other three types of implants (Fig. 4). In the maxilla, PIII-PEEK-FFF implants showed significantly higher BA/TA compared to untreated control PEEK-FFF implants group in the 24–80 μm zone (Fig. 5).

On multivariable random effects analysis for mandibular implants, after adjusting for the effect of the location and time, the PIII-PEK-SLS implant had significantly higher mean BA/TA ratio compared to the untreated control PEEK-FFF implant in the 0–24 μm zone (b = 0.42, *p* < 0.001) and 24–80 μm zone (b = 0.27, *p* = 0.001). BA/TA ratio in PIII-PEK-SLS implant group was also significantly higher than that of PIII-PEEK-FFF implant group in the 0–24 μm zone (b = 0.25, *p* = 0.019). Similar estimates were found for the titanium positive control implant in the 0–24 μm zone (b = 0.34, *p* < 0.001) and 24–80 μm zone (b = 0.28, *p* = 0.001). The PIII-PEEK-FFF implant also had significantly higher mean BA/TA ratio compared to the untreated control PEEK-FFF implant in the 24–80 μm zone (b = 0.22, *p* < 0.001), however, this was less than that observed for PIII-PEK-SLS and titanium.

## Discussion

Bone contact with the implant surface is important for the long-term success of implanted prostheses, especially in load-bearing maxillofacial reconstruction^23^. The current pilot ovine study was conducted with the aim of evaluating the in vivo osseointegration properties of surgically implanted plasma-treated PEEK and PEK cylindrical implants with a saw-tooth surface structure. It is commonly recognized that implant hydrophilicity and surface topology play an essential role in osseointegration^24^. The macro and micro surface roughness can enhance the attachment of bone tissues onto implant surfaces^25^.

Osseointegration was evaluated qualitatively and quantitatively using histological analysis of BIC in two zones adjacent to the saw-tooth surface, namely 0–24 μm and 24–80 μm. As shown by the BIC analysis, plasma treated PEEK-FFF and PEK-SLS demonstrated superior osseointegration to untreated control PEEK-FFF implants. SEM and AFM examination revealed the PEK-SLS has increased macro and micro surface roughness compared to PEEK-FFF implants, enabling better interlocking with the bone at its surface, resulting in significantly better BIC.

Qualitative assessment of the Goldner’s trichome stained mandibular bone-implant tissue sections from PIII-treated PEEK-FFF and PEK-SLS experimental groups suggested that the intersectional space between the implant surface and the surrounding bone was richer in mineralized bone tissue towards the end point of the study. In contrast, in the untreated control PEEK-FFF group, the space was filled predominantly with collagen-rich connective tissues. These qualitative observations suggest better integration between the implant and bone in the experimental groups compared to the untreated negative controls, and similar to that observed in the titanium positive control.

Our findings suggest that osseointegration is facilitated by plasma treatment of the PEEK implants surface. Although the untreated control, PEEK-FFF, is hydrophobic, high energy PIII-treatment modifies the surface structure of the material long-term by creating free radicals that form covalent bonds with adjacent proteins, thus enhancing hydrophilicity and enabling cellular attachment, migration, and tissue growth^26^. This finding is further supported by recent in vitro work where PIII-treated PEEK-FFF mesh scaffolds were infilled with Saos-2 osteoblast‐like cells and cultured with protein-enriched media. The hydrophilic surface of the PIII-treated PEEK-FFF scaffold formed covalent bonds with the amorphous calcium phosphate‐associated protein (ACPAP) and showed improved cellular adhesion, rapid proliferation and secretion of extracellular matrix protein, mediating the deposition of calcium and phosphate into the extracellular matrix, forming ACPAP^21,27^.

Moreover, PIII-treated PEEK has recently been reported to demonstrate better osteoconduction compared to untreated PEEK^28^. Whilst there are other surface modification strategies, including wet chemistry and titanium coating^29–32^, these techniques require further intensive research to reduce the toxicity of de-bonded nanoparticles and long-term failure from delamination. In contrast, the PIII surface modification technique does not require wet chemistry, simplifying the bioengineering process and facilitating clinical translation^21^.

Quantitative analysis of the BIC showed comparable osseointegration of the SLS-printed PEK implants compared titanium implants in each zone (0–24 μm and 24–80 μm) at each time point (8-, 10-, and 12-weeks) of the study. Furthermore, the PIII-treated FFF-printed PEEK implants showed significantly better osseointegration than the untreated FFF-printed PEEK implants, which revealed a lower BIC compared to all other implant tested across all time points in both BIC zones. These results were consistent in both the mandible and maxilla, suggesting enhanced osseointegration mediated by plasma treatment. Previous studies support these findings, showing that untreated PEEK provides less bony integration compared to that of titanium implants, with the space between the untreated PEEK and the native bone being filled with fibrous tissue^33^. Interestingly, untreated PEEK implants are more prone to bacterial infection compared to the surface-modified PEEK, which may also reduce osseointegration^34^. In contrast, PEEK implants treated with various surface modification techniques are associated with improved osseointegration^19,35–38^.

A recent in vivo ovine study conducted by our group employed FFF-printed PEEK discs designed for mechanical assessment of osseointegration using a torque test revealed that higher forces were required to break the surface bond between PIII-treated PEEK-FFF compared to untreated control PEEK-FFF implants^39^. Surface treatment of the PEEK using various techniques enhances its bioactivity without changing the chemistry and mechanical properties of PEEK^40^. Untreated PEEK facilitates fibrous tissue formation^41^, which is not favorable for strong bone-implant contact.

Furthermore, untreated PEEK compared to plasma treated PEEK, is associated with less mineralisation^42^. Similarly, both qualitative and quantitative histological evaluations for the present study showed better mineralization and bone-implant contact for PIII-treated PEEK-FFF and PEK-SLS implants, suggesting the efficiency of PIII surface modification for improved osseointegration.

The main limitation of this study is the small sample size. Whilst we aimed to have a minimum of six samples of each implant type, this was not possible due to the need to euthanise one sheep due to aspiration of stomach contents. The exclusion of this sheep may have introduced skewed results, particularly if the omitted data would have shown divergent outcomes. This highlights the critical importance of maintaining a sufficiently large sample size in future investigations to mitigate the impact of such losses and uphold the integrity of the study.

There are several variables that may have had a confounding effect, including differences in primary stability, bone quality, and bone thickness at the site of implantation. Primary stability of bone implants play a vital role in establishing strong bone-implant contact at insertion^43^. Primary stability of the implant is determined by various factors, including bone volume and density, implant design, manufacturing procedure, and surgical technique^43^. In this study, we eliminated primary stability as a confounding variable by making the osteotomy larger than the implant. Hence, there was minimal, if any, bone-implant contact at insertion.

Finally, whilst this study clearly demonstrates that PIII-treatment improves the osseointegration of FFF-printed PEEK, it is unclear whether the superior performance of the SLS printed PEK implants was due to the PIII treatment, the surface topology, and/or material properties.

The results from this study suggest that PIII-treatment enhances the bioactivity of FFF printed PEEK implants and that SLS printed PEK implants demonstrate similar osseointegration to 3D-printed titanium implants of the same design. The enhanced biocompatibility and mechanical properties of these materials closely resemble natural bone, ensuring better load distribution and reducing implant failure risks. Additionally, the radiolucency feature of these biomaterials allows for enhanced post-operative imaging, crucial for monitoring tumor recurrence and implant integration. Improved surface roughness and energy from PIII treatment promote superior osseointegration, enhancing implant stability and integration. Combined with 3D printing, PIII-treated PEEK and PEK can be customized to fit patient-specific anatomical needs, offering tailored and effective solutions for complex reconstructive challenges in cancer care and better match the mechanical properties of bone than that of titanium. Further preclinical research on the application of PIII-treated PEEK and PEK as bone scaffolds or implants in repairing segmental mandibular defects, both with and without osteoinductive components such as osteogenic cells, is essential for gaining deeper insights and advancing their clinical translation.

## Methods

**Fig. 6:**
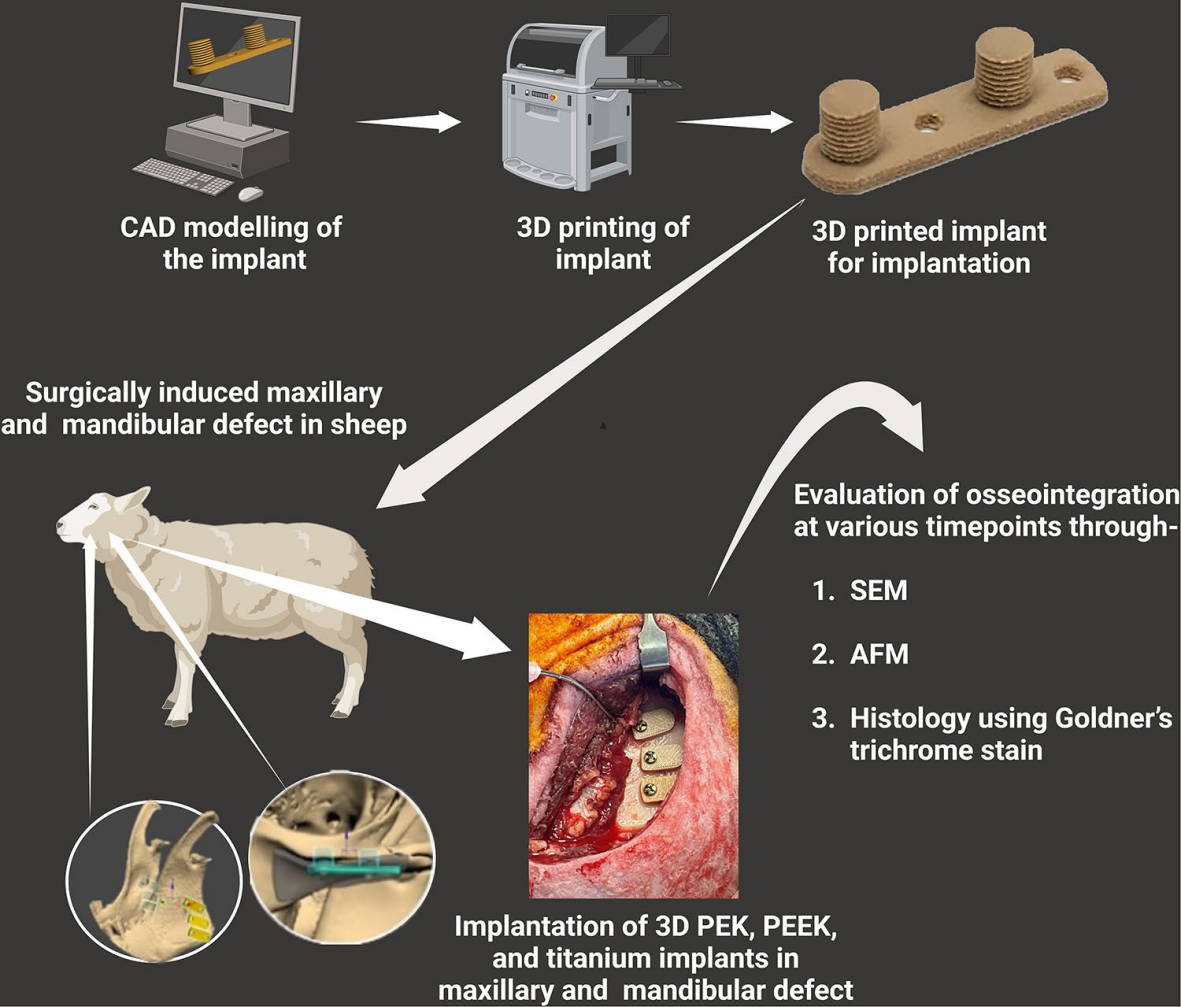
Overview of the general methodology employed in this study.

The schematic in Fig. 6 outlines the steps involved in designing, printing, and evaluating 3D-printed implants for osseointegration in a sheep model with surgically induced maxillary and mandibular defects. The process begins with CAD modelling of the implant, followed by 3D printing and implantation in defect sites created surgically. The osseointegration of the implanted 3D-printed PEK, PEEK, and titanium implants is then evaluated over time using scanning electron microscopy (SEM), atomic force microscopy (AFM), and histological staining with Goldner’s trichrome. All procedures adhered to the guidelines and regulations concerning the care and utilisation of animals in research, as approved by the animal ethics committee at the University of Sydney, Australia (ethics approval number: 2020/1817). Additionally, compliance with ARRIVE (Animal Research: Reporting In Vivo Experiments) guidelines was meticulously maintained.

## Implant design

The design of mandible and maxilla implants utilised 3dsMax 2020 (Autodesk, Inc., San Francisco, California, U.S.) and the polygonal modeling technique. The modeling was performed on an Alienware A51 v1.12 workstation (DELL Inc.) equipped with an Intel Core i7-9700 CPU (3.00 GHz, 8 Core(s)), 64 GB RAM, and an NVIDIA GeForce RTX 2080 graphics card (NVIDIA Corporation, Santa Clara, California, U.S.).

The mandible implant is characterized by a single cylinder measuring 5 mm in height and diameter, featuring nine grooves that create a ‘saw-tooth’ structure to enhance surface area, incorporating both single and double fixation sites (Supplementary Fig. 1A). For the maxillary implants (Supplementary Fig. 1B), two cylinders and two fixation sites were employed. The digital implant models for the right mandible and maxilla were generated using the mirror function, mirroring the left side of the mandible implant and maxillary implant in 3D.

### Additive manufacturing of 3D PEEK implant

The PEEK mandible and maxilla implants were manufactured through fused filament fabrication (FFF) on an AON-M.2 3D printer (firmware v3.3.5) from AON3D in Montreal, Canada. The printing utilized 1.75 mm Thermax PEEK (batch 49-080620-06JV) filament produced by 3DXTech in Grand Rapids, Michigan. A 0.4 mm diameter E3D-V6 Nozzle X and a high-temperature polyetherimide (PEI) build plate were employed for platform adherence. Before printing, the filament spool underwent an 8-hour drying process at 120℃ and was stored in a sealed filament box at 10% relative humidity during printing. Z calibration was performed before each print to ensure inter-batch consistency. Designs were oriented with the flat base in contact with a raft, as depicted in Supplementary Fig. 2, eliminating the need for support material and potential contamination to the implant from the build plate. The PEEK implant designs were sliced for 3D printing using Simplify3D (V4.1.2) (Supplementary Fig. 2). Key printing parameters are detailed in Supplementary Table 1. To achieve consistent definition of the saw-tooth structures, perimeters of each layer were printed from inner to outer, and the printing layer thicknesses were matched to an integer division of the groove feature sizes. PEEK processing involved high temperatures in the nozzle, platform, and chamber to target high polymer crystallinity, confirmed by the implants’ opaque light-beige colour.

The PEK implants were produced using selective laser sintering (SLS) on an EOS P800 (EOS GmbH, Krailling, Germany) and EOS PEEK HP3 as the feedstock material. Designs were printed with the cylinder axis aligned to the Z-axis, and the cylinder side faced downwards. Layers were sliced with a thickness of 0.12 mm using Materialise Magics (software version 23.0.1.19) and the EOS build processor (version 1.2). Layer files were assigned the EOS_dec laser exposure strategy from the PAEK1304_120_011 material set using the EOS PSW software (version 3.7). After completion of the build process, the implants were allowed to cool with the default high-temperature cooldown routine until temperatures fell below 60℃. Subsequently, implants were removed from the powder cake and cleaned with dry ice using a ColdJet MicroClean at 4.2 bar gauge pressure and a feed rate of 0.15 kg/min.

## Additive manufacturing of 3D Titanium implant

Titanium mandible implants with double fixation sites were manufactured using a powder bed fusion technique known as selective laser melting (SLM). The body of work employed Grade 23 titanium alloy (CL41 Ti6Al4V-ELI, 20–63 μm, AP&C, Canada) powder and a Concept Laser MLAB200R (GE Additive, Boston, United States) metal additive manufacturing system. STL files, featuring the same geometry discussed earlier, were pre-processed using Materialise Magics software version 21 with the Concept Laser MLAB200R build volume profile. The files were oriented with the axis of the implant cylinder aligned to the Z-axis of the build direction, with the cylinder side facing upwards, providing the most reliable orientation for accurately recreating the saw-tooth structure of the implant. The implant’s base was offset by 3 mm from the build platform. Support structures were applied to secure the implant to the build platform during processing and to conduct heat away from weld zones during printing. The combined model and support files were then transferred to the Concept Laser MLAB200R workstation, where slice and lasing parameters were applied according to the manufacturer-supplied material parameter file (Mlab 200R Titanium Ti6Al4V Base Parameter Profile 0012, GE Additive), specifying a layer height of 25 μm.

After printing, components were removed from the inert process chamber, excess powder was removed, and parts were manually separated from the build tray using side cutters. The roughened interface where the implant’s base met the support structure was cleaned and finished using 200-grit sandpaper on a belt linisher. Implant surfaces underwent further processing to ensure the removal of all loosely bound powder particles through manual media blasting (Ø20 µm glass bead, Peenmatic 550, Iepco AG, Höri, Switzerland) at 2.0 bar gauge pressure.

## PIII treatment of PEEK and PEK implants

The operating principle of the PIII process has been described in detail previously^44^. For the present study, the FFF-printed PEEK and SLS-printed PEK implants were placed in a borosilicate glass 250 ml Erlenmeyer flask and evacuated to a base pressure of lower than 20 mTorr. PIII treatment was carried out by immersion in a di-electric barrier discharge in nitrogen gas at two different pressures of 350 and 700 mTorr. The discharge was excited through a copper foil electrode, covering the bottom and the sides of the treatment vessel up to a height of 10 mm. Negative voltage pulses of 10 kV were applied to the electrode using an RuP6 switch mode power supply (GBS Elektronik GmbH, Radeberg, Germany). The pulse frequency was set at 1000 Hz, the pulse length at 40 µs, and the total treatment time was 20 min at each pressure. After PIII treatment, all implants underwent steam sterilization before being implanted into the sheep mandible and maxilla.

### SEM and AFM assessment of PIII treated PEEK and PEK implants

Implants were sectioned and subjected to sputter-coating with platinum before being imaged using a TESCAN MIRA3 SEM (Tescan, Czech Republic). The examination of surface roughness was conducted using a Dimension Icon AFM (Bruker, Massachusetts, U.S), and the results were presented as an image of Ra, representing the arithmetic average of the absolute values of the surface height deviations measured from the mean plane.

### Implantation surgery

Six female sheep, aged between 7 and 8 years, and weighing 70–80 kg each, were divided into three groups, with two sheep per group, representing distinct timepoints (8-, 10-, and 12-weeks post-implantation, as detailed in Supplementary Table 2). The timepoints were selected based on existing literature from similar animal studies evaluating osseointegration of various bone and dental implants^45–48^. Prior to surgery, the sheep were housed on straw bedding and maintained on a standard chaff and hay diet for at least two weeks. A thorough physical examination confirmed the health of all animals before the surgical procedures.

The sheep underwent premedication with 0.2–0.5 mg/kg methadone (Methodyne^®^, Jurox, Australia) and 0.2–0.5 mg/kg diazepam (Ilium diazepam, Troy, Australia) through a pre-placed intravenous (IV) cannula. General anaesthesia was induced with IV propofol (Propofol-Lipuro 1%, B. Braun Melsungen AG, Germany) at a dosage of 2–4 mg/kg. Following induction, the entire head region was shaved, and surgical sites were sterilised using chlorhexidine. A 10 cm incision was made along the lower border of the right and left mandible. The masseter muscle was stripped off the lateral ramus of the mandible, and three full-thickness osteotomies were created using a drill guide and sequential dental drills (Southern Implants Pty Ltd, 1 Albert Road, Irene, RSA).

**Fig. 7:**
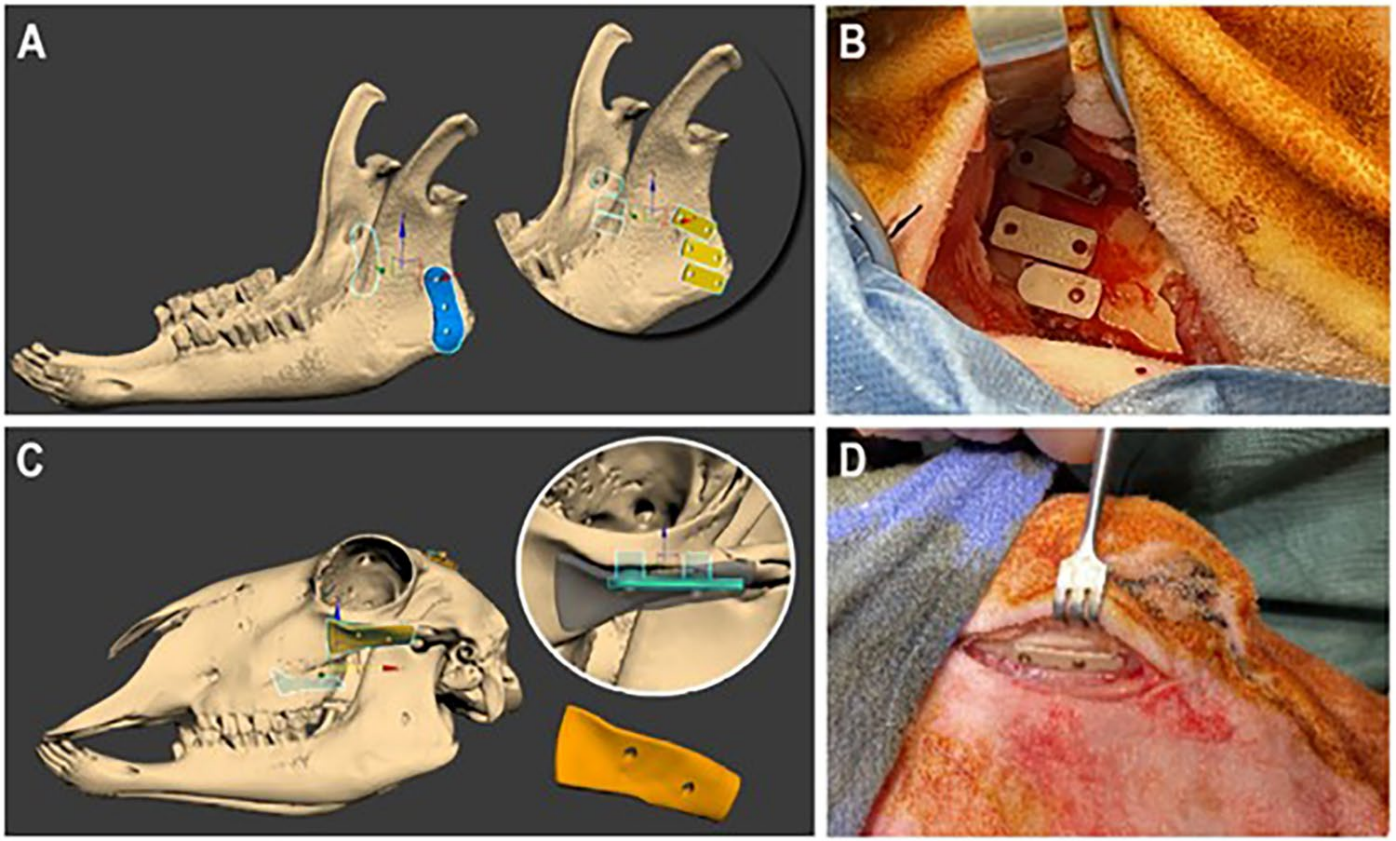
Implantation sites in mandible and maxilla. (**A**) The mandibular implantation site: Two PIII-PEEK-FFF and one PIII-PEK-SLS implants were placed on the left side and one titanium and one untreated PEEK-FFF implants were placed on the right side. (**B**) Surgical implantation of PIII-PEEK-FFF mandibular implants. (**C**) The maxillary implantation site: One double PIII-PEEK-FFF implant was placed on the left side and one double untreated PEEK-FFF implant was placed on the right side. (**D**) Surgical implantation of a double PEEK-FFF maxillary implant.

Osteotomies, 0.6 mm over-sized to prevent damage to the implant’s saw-tooth structure and allow for any offset, were made using low-speed irrigation. Two PIII treated PEEK-FFF implants (superior and middle) and a single PIII treated PEK-SLS implant (inferior) were passively placed in the left mandibular vertical ramus. In the right mandible, a titanium implant (superior) and an untreated PEEK-FFF implant (inferior) were placed as controls (Fig. 7A and B). For maxilla implantation, incisions along the zygomatic arch were made bilaterally, and a similar procedure was followed (Fig. 7C and D). PIII treated PEEK-FFF double implants were placed in the left maxilla, while untreated control PEEK-FFF double implants were placed in the right maxilla. All implants were secured to the native bone using 2 mm x 6 mm titanium fixation screws. Intraoperative cone beam CT (Siemens Artis Pheno, Siemens Healthcare GmbH, Erlangen, Germany) confirmed implant locations. Wounds were closed in two layers using 4/0 absorbable suture composed of 90% glycolide and 10% L-lactide (Vicryl^®^, Johnson & Johnson, North Ryde, NSW, Australia) for deep tissue and 4/0 absorbable suture composed of Poliglecaprone 25 (Monocryl^®^, Johnson & Johnson, North Ryde, NSW, Australia) for the skin. Betadine ointment was applied for dressing.

Unfortunately, one sheep from group 2 (the 10-week implantation group) was euthanised on the surgery day due to aspiration. Euthanasia for the remaining sheep in group 1, group 2, and group 3 was performed at the end of 8-, 10-, and 12-weeks after the surgery, respectively (Sheep were euthanised after placement of a cephalic intravenous cannula. Propofol 4 mg/kg was administered intravenously to induce unconsciousness followed by sodium pentobarbitone 150 mg/kg IV to implement euthanasia). All samples were harvested at each timepoint and immediately stored in 10% neutral buffered formalin to fix the tissue for subsequent histological analysis.

### Histology

#### Resin embedding and sectioning

All sheep explant samples underwent trimming with an EXAKT precision band saw, aiming to eliminate excess bone tissues surrounding the implants’ plates. This process left 2–5 mm of peripheral bone tissue around each individual implant, preparing the samples for analysis using resin histology. As previously documented^49,50^, the samples underwent dehydration with increasing grades of ethanol and were subsequently infiltrated and embedded in a Technovit 9100 methyl methacrylate system (Kulzer GmbH, Wehrheim, Germany) without decalcification. Resin ground sections, approximately 50 μm in thickness, were obtained on standard microscope slides using an EXAKT cutting and grinding system (EXAKT Advanced Technologies GmbH, Norderstedt, Germany).

### Goldner’s trichrome stain

The ground sections underwent staining using Goldner’s trichrome following published methods^50^. Briefly, the sections were initially stained with Weigert’s haematoxylin (Merck, Bayswater, VIC, Australia) for 25 min, followed by washing and immersion in the acid Fuchsin-Ponceau working solution (Fuchsin, Merck, Bayswater, VIC, Australia) for 10 min. After washes in 1% acetic acid, the sections were subjected to staining with tungstophosphoric acid - orange G Fuchsin solution (Merck, Bayswater, VIC, Australia) for 20 min and light green solution for 15 min. Subsequently, the sections were air-dried, cleared in xylene, and mounted for imaging.

### Imaging

Imaging of implants was conducted using a Zeiss Observer 7 microscope (Carl Zeiss Microscopy, Germany) at x5 magnification, employing brightfield tile acquisition mode. The tiled images capturing the entire implants were stitched together and exported in TIFF format for subsequent analysis.

### Histomorphometry of bone-implant contact analysis

**Fig. 8:**
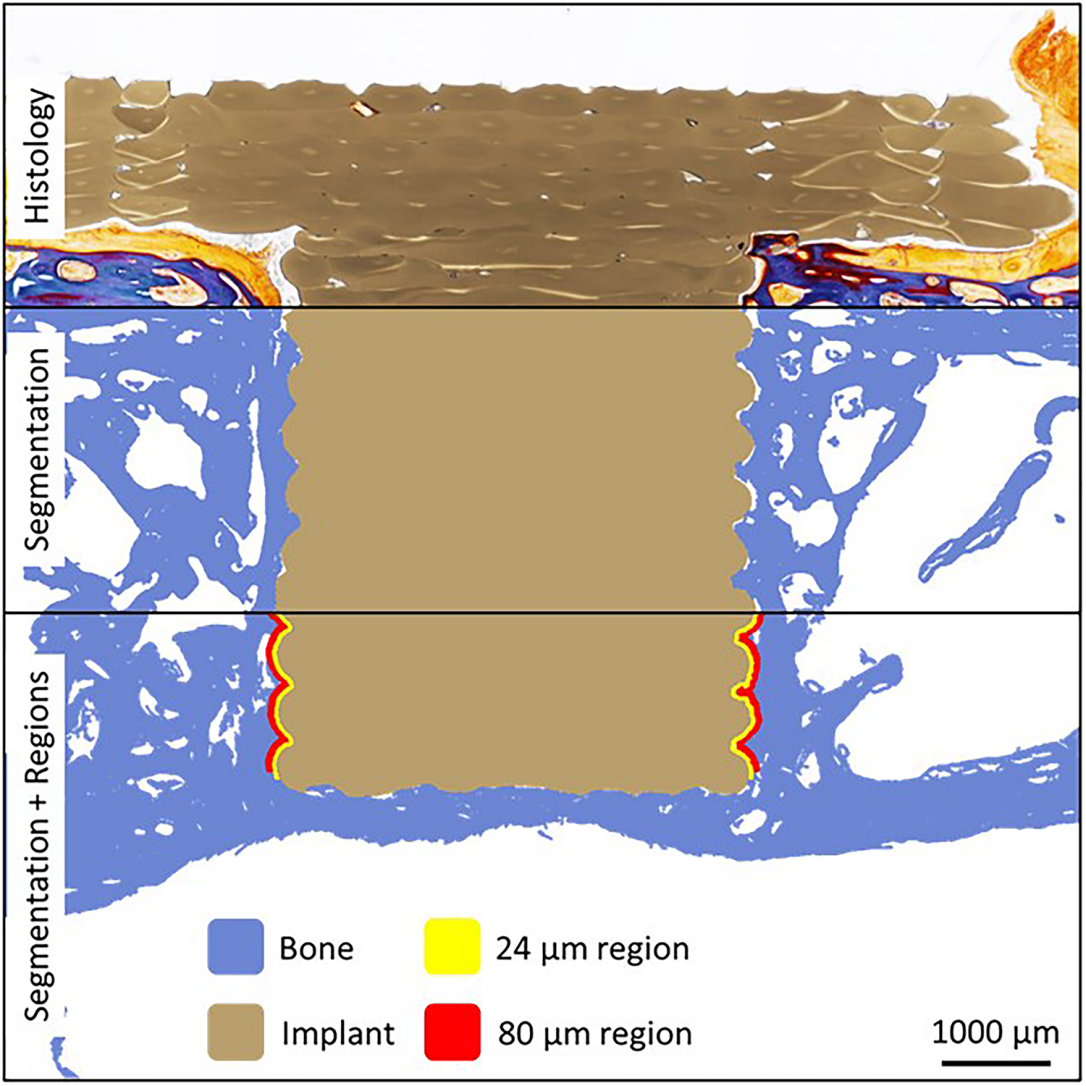
Illustration of histomorphometric analysis of BIC using an example histology image. Two regions were selected for analysis based on their distance from the implant surface: 0–24 μm and 24–80 μm. The image was segmented to distinguish the implant, bone matrix, and the two zones of BA/TA quantification.

BIC was quantified through histomorphometry image processing of the histology images using a custom code (MATLAB 2021a), following a methodology similar to that of Butz et al. (2006)^51^. In brief, the bone and implant regions were segmented using manual thresholding in the L*a*b* colour space. Measurements were specifically carried out at the implant thread (or saw-tooth) by masking and excluding the implant head and shank. Osseointegration was assessed based on the bone area (BA) to total area (TA) ratio (BA/TA) within two regions, namely 24 μm and 80 μm from the implant thread^52^. Figure 8 offers a diagrammatic representation of this process, illustrating the histological micrograph, segmentation, and the 24 μm and 80 μm regions.

### Statistical analysis

Statistical analysis was performed using Stata Statistical Software: Release 17 (StataCorp LLC, College Station TX, USA) and GraphPad Prism 9 software (GraphPad Software Inc, San Diego CA, USA). A total of 16 samples per group were required to detect a difference in BA/TA ratio of 0.50 with a standard deviation of 0.5, 80% power, and alpha = 0.05. The differences in BA/TA data obtained by histology image quantification between implant groups (PIII-treated PEEK *n* = 20; PIII-treated PEK *n* = 5; Untreated PEEK *n* = 15; Titanium *n* = 5) were compared using one-way ANOVA (Tukey’s multiple comparisons test) and multivariable analyses of all 46 implants combined were performed using random effects modelling to account for correlated data adjusting for the effect of time and location. All analyses were two sided and *p* < 0.05 was considered statistically significant.

## Electronic supplementary material

Below is the link to the electronic supplementary material.

Supplementary Material 1

Supplementary Material 2

Supplementary Material 3

## Data Availability

The datasets generated during and/or analysed during the current study are available from the corresponding author on reasonable request.
