# Supplementary figures and images for: Evaluation of osseointegration of plasma treated polyaryletherketone maxillofacial implants
Source: Sci Rep. 2025 Jan 13;15:1895. doi: 10.1038/s41598-024-80335-z (PMC11731023; doi:10.1038/s41598-024-80335-z)

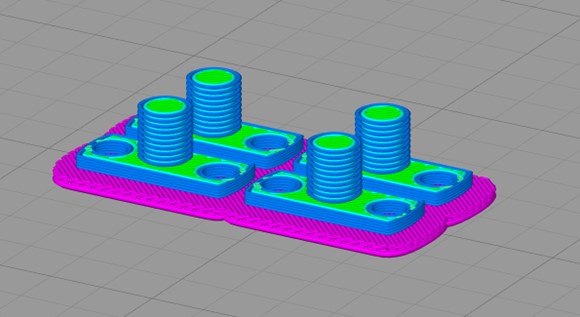

Supplement: Supplementary file 1 — Supplementary Material 1 [file 41598_2024_80335_MOESM1_ESM.jpg]

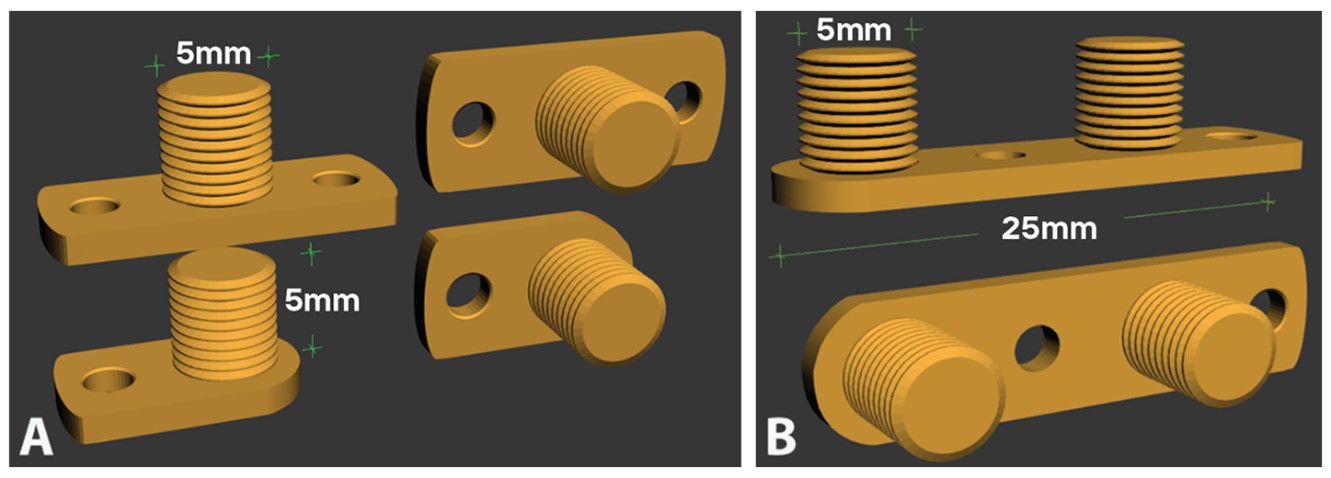

Supplement: Supplementary file 2 — Supplementary Material 2 [file 41598_2024_80335_MOESM2_ESM.jpg]
