# Supplementary material for: Evaluation of osseointegration of plasma treated polyaryletherketone maxillofacial implants
Source: Sci Rep. 2025 Jan 13;15:1895. doi: 10.1038/s41598-024-80335-z (PMC11731023; doi:10.1038/s41598-024-80335-z)
Supplement: Supplementary file 3 — Supplementary Material 3 [file 41598_2024_80335_MOESM3_ESM.docx]

Supplementary Figure 1. Implant designs. (A) Design of mandible implants with single cylinder with single and double fixation points; (B) Design of maxillary implants with double cylinders with double fixation points.

Supplementary Figure 2. The slice preview of the grommet implants generated by Simplify3D software. The preview demonstrates the printing layers and print orientation with respect to the build plate (grey grid). It also shows the removable raft (pink), as well as the different building features of the implant; solid infill (green), inner perimeter (light blue) outer perimeter (dark blue).

Supplementary Table 1: FFF PEEK 3D Printing Parameters

| Nozzle temperature | 405°C |
| --- | --- |
| Platform temperature | 170°C |
| Chamber temperature | 120°C |
| Layer height | 0.25 mm |
| Extrusion width | 0.5 mm |
| Print speed | 30 mm/s |
| Outline underspeed | 70% |
| Shells | 2 |
| Infill percentage | 100% |
| Infill pattern: raster angles | Rectilinear: 45°, -45° |
| Raft | 3 layers of PEEK, 100% density |

Supplementary Table 2: Location and number of implants at different timepoints available for analysis.

|  | **Group 1 (8 weeks)** | | | | **Group 2 (10 weeks)** | | | | **Group 3 (12 weeks)** | | | |
| --- | --- | --- | --- | --- | --- | --- | --- | --- | --- | --- | --- | --- |
|  | **Mandible**  **(ramus)** | | | **Maxilla (zygoma)** | **Mandible**  **(ramus)** | | | **Maxilla (zygoma)** | **Mandible**  **(ramus)** | | | **Maxilla (zygoma)** |
|  | **Superior** | **Middle** | **Inferior** |  | **Superior** | **Middle** | **Inferior** |  | **Superior** | **Middle** | **Inferior** |  |
| **PIII-treated PEEK (n = 20)** | 2 | 2 | 0 | 4 | 1 | 1 | 0 | 2 | 2 | 2 | 0 | 4 |
| **PIII-treated PEK (n = 5)** | 0 | 0 | 2 | 0 | 0 | 0 | 1 | 0 | 0 | 0 | 2 | 0 |
| **Untreated PEEK**  **(n = 15)** | 0 | 0 | 2 | 4 | 0 | 0 | 1 | 2 | 0 | 0 | 2 | 4 |
| **Titanium**  **(n = 6)** | 2 | 0 | 0 | 0 | 1 | 0 | 0 | 0 | 2 | 0 | 0 | 0 |
|  |  |  |  |  |  |  |  |  |  |  |  |  |

*Note: One sheep in group 2 (10 week) was euthanised due to aspiration. Implants placed in the mandible were single and maxilla were double.*
